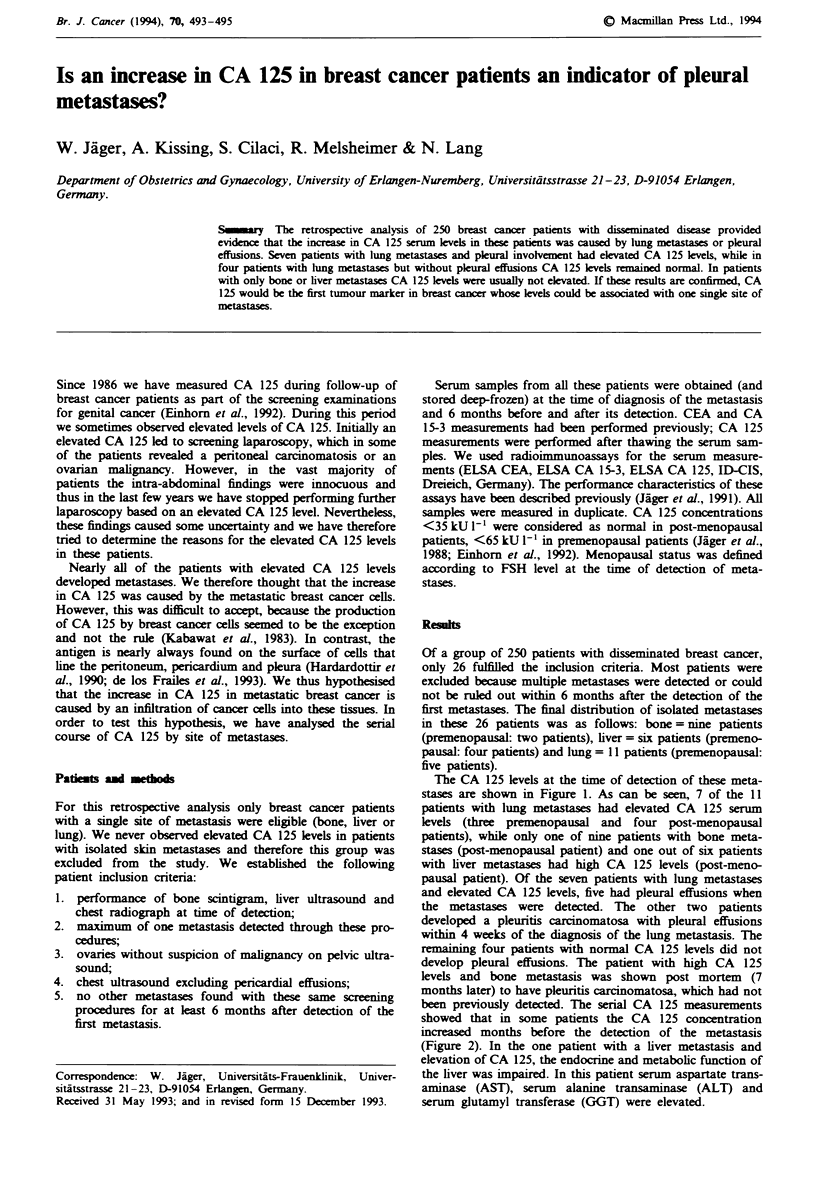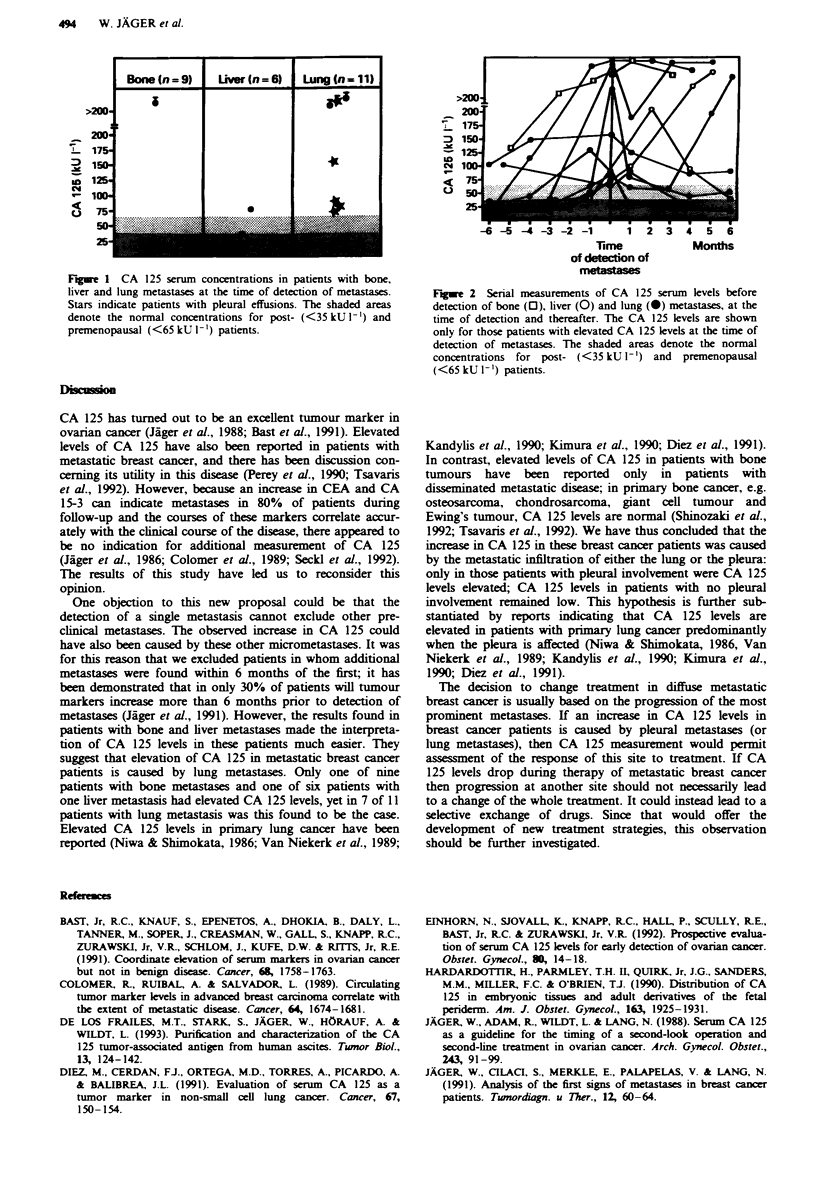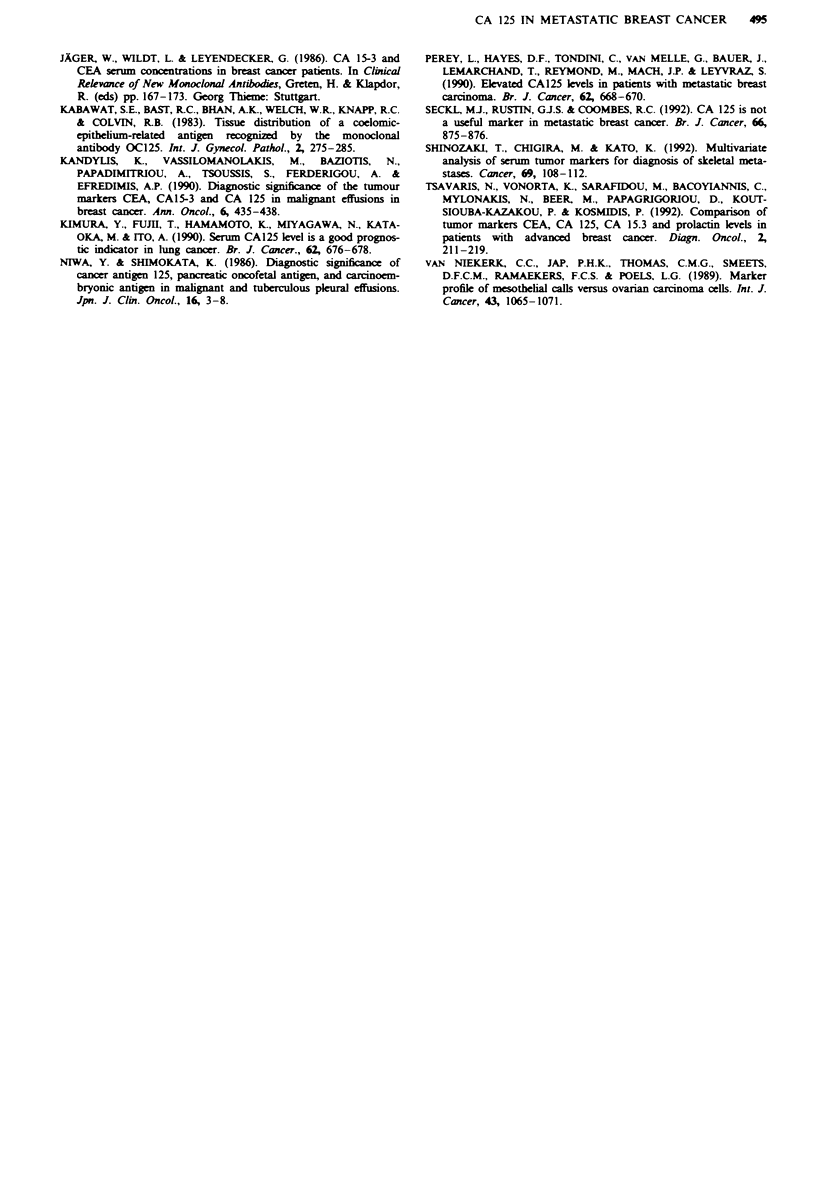# Is an increase in CA 125 in breast cancer patients an indicator of pleural metastases?

**DOI:** 10.1038/bjc.1994.333

**Published:** 1994-09

**Authors:** W. Jäger, A. Kissing, S. Cilaci, R. Melsheimer, N. Lang

**Affiliations:** Department of Obstetrics and Gynaecology, University of Erlangen-nuremberg, Germany.

## Abstract

The retrospective analysis of 250 breast cancer patients with disseminated disease provided evidence that the increase in CA 125 serum levels in these patients was caused by lung metastases or pleural effusions. Seven patients with lung metastases and pleural involvement had elevated CA 125 levels, while in four patients with lung metastases but without pleural effusions CA 125 levels remained normal. In patients with only bone or liver metastases CA 125 levels were usually not elevated. If these results are confirmed, CA 125 would be the first tumour marker in breast cancer whose levels could be associated with one single site of metastases.


Br. J. Cancer (1994), 70, 493–495 © Macmillan Press Ltd., 1994

### Is an increase in CA 125 in breast cancer patients an indicator of pleural metastases?

W. Jäger, A. Kissing, S. Cilaci, R. Melsheimer & N. Lang

*Department of Obstetrics and Gynaecology, University of Erlangen-Nuremberg, Universitätsstrasse 21–23, D-91054 Erlangen, Germany.*

**Summary** The retrospective analysis of 250 breast cancer patients with disseminated disease provided evidence that the increase in CA 125 serum levels in these patients was caused by lung metastases or pleural effusions. Seven patients with lung metastases and pleural involvement had elevated CA 125 levels, while in four patients with lung metastases but without pleural effusions CA 125 levels remained normal. In patients with only bone or liver metastases CA 125 levels were usually not elevated. If these results are confirmed, CA 125 would be the first tumour marker in breast cancer whose levels could be associated with one single site of metastases.

Since 1986 we have measured CA 125 during follow-up of breast cancer patients as part of the screening examinations for genital cancer (Einhorn *et al.*, 1992). During this period we sometimes observed elevated levels of CA 125. Initially an elevated CA 125 led to screening laparoscopy, which in some of the patients revealed a peritoneal carcinomatosis or an ovarian malignancy. However, in the vast majority of patients the intra-abdominal findings were innocuous and thus in the last few years we have stopped performing further laparoscopy based on an elevated CA 125 level. Nevertheless, these findings caused some uncertainty and we have therefore tried to determine the reasons for the elevated CA 125 levels in these patients.

Nearly all of the patients with elevated CA 125 levels developed metastases. We therefore thought that the increase in CA 125 was caused by the metastatic breast cancer cells. However, this was difficult to accept, because the production of CA 125 by breast cancer cells seemed to be the exception and not the rule (Kabawat *et al.*, 1983). In contrast, the antigen is nearly always found on the surface of cells that line the peritoneum, pericardium and pleura (Hardardottir *et al.*, 1990; de los Frailes *et al.*, 1993). We thus hypothesised that the increase in CA 125 in metastatic breast cancer is caused by an infiltration of cancer cells into these tissues. In order to test this hypothesis, we have analysed the serial course of CA 125 by site of metastases.

### Patients and methods

For this retrospective analysis only breast cancer patients with a single site of metastasis were eligible (bone, liver or lung). We never observed elevated CA 125 levels in patients with isolated skin metastases and therefore this group was excluded from the study. We established the following patient inclusion criteria:

1. performance of bone scintigram, liver ultrasound and chest radiograph at time of detection;
2. maximum of one metastasis detected through these procedures;
3. ovaries without suspicion of malignancy on pelvic ultrasound;
4. chest ultrasound excluding pericardial effusions;
5. no other metastases found with these same screening procedures for at least 6 months after detection of the first metastasis.

Correspondence: W. Jäger, Universitäts-Frauenklinik, Universitätsstrasse 21–23, D-91054 Erlangen, Germany.
Received 31 May 1993; and in revised form 15 December 1993.

Serum samples from all these patients were obtained (and stored deep-frozen) at the time of diagnosis of the metastasis and 6 months before and after its detection. CEA and CA 15-3 measurements had been performed previously; CA 125 measurements were performed after thawing the serum samples. We used radioimmunoassays for the serum measurements (ELSA CEA, ELSA CA 15-3, ELSA CA 125, ID-CIS, Dreieich, Germany). The performance characteristics of these assays have been described previously (Jäger *et al.*, 1991). All samples were measured in duplicate. CA 125 concentrations $<35\ kU\ l^{-1}$ were considered as normal in post-menopausal patients, $<65\ kU\ l^{-1}$ in premenopausal patients (Jäger *et al.*, 1988; Einhorn *et al.*, 1992). Menopausal status was defined according to FSH level at the time of detection of metastases.

### Results

Of a group of 250 patients with disseminated breast cancer, only 26 fulfilled the inclusion criteria. Most patients were excluded because multiple metastases were detected or could not be ruled out within 6 months after the detection of the first metastases. The final distribution of isolated metastases in these 26 patients was as follows: bone = nine patients (premenopausal: two patients), liver = six patients (premenopausal: four patients) and lung = 11 patients (premenopausal: five patients).

The CA 125 levels at the time of detection of these metastases are shown in Figure 1. As can be seen, 7 of the 11 patients with lung metastases had elevated CA 125 serum levels (three premenopausal and four post-menopausal patients), while only one of nine patients with bone metastases (post-menopausal patient) and one out of six patients with liver metastases had high CA 125 levels (post-menopausal patient). Of the seven patients with lung metastases and elevated CA 125 levels, five had pleural effusions when the metastases were detected. The other two patients developed a pleuritis carcinomatosa with pleural effusions within 4 weeks of the diagnosis of the lung metastasis. The remaining four patients with normal CA 125 levels did not develop pleural effusions. The patient with high CA 125 levels and bone metastasis was shown post mortem (7 months later) to have pleuritis carcinomatosa, which had not been previously detected. The serial CA 125 measurements showed that in some patients the CA 125 concentration increased months before the detection of the metastasis (Figure 2). In the one patient with a liver metastasis and elevation of CA 125, the endocrine and metabolic function of the liver was impaired. In this patient serum aspartate transaminase (AST), serum alanine transaminase (ALT) and serum glutamyl transferase (GGT) were elevated.

**Figure 1** CA 125 serum concentrations in patients with bone, liver and lung metastases at the time of detection of metastases. Stars indicate patients with pleural effusions. The shaded areas denote the normal concentrations for post- (<35 kU l$^{-1}$) and premenopausal (<65 kU l$^{-1}$) patients.

**Figure 2** Serial measurements of CA 125 serum levels before detection of bone (□), liver (○) and lung (●) metastases, at the time of detection and thereafter. The CA 125 levels are shown only for those patients with elevated CA 125 levels at the time of detection of metastases. The shaded areas denote the normal concentrations for post- (<35 kU l$^{-1}$) and premenopausal (<65 kU l$^{-1}$) patients.

### Discussion

CA 125 has turned out to be an excellent tumour marker in ovarian cancer (Jäger et al., 1988; Bast et al., 1991). Elevated levels of CA 125 have also been reported in patients with metastatic breast cancer, and there has been discussion concerning its utility in this disease (Perey et al., 1990; Tsavaris et al., 1992). However, because an increase in CEA and CA 15-3 can indicate metastases in 80% of patients during follow-up and the courses of these markers correlate accurately with the clinical course of the disease, there appeared to be no indication for additional measurement of CA 125 (Jäger et al., 1986; Colomer et al., 1989; Seckl et al., 1992). The results of this study have led us to reconsider this opinion.

One objection to this new proposal could be that the detection of a single metastasis cannot exclude other preclinical metastases. The observed increase in CA 125 could have also been caused by these other micrometastases. It was for this reason that we excluded patients in whom additional metastases were found within 6 months of the first; it has been demonstrated that in only 30% of patients will tumour markers increase more than 6 months prior to detection of metastases (Jäger et al., 1991). However, the results found in patients with bone and liver metastases made the interpretation of CA 125 levels in these patients much easier. They suggest that elevation of CA 125 in metastatic breast cancer patients is caused by lung metastases. Only one of nine patients with bone metastases and one of six patients with one liver metastasis had elevated CA 125 levels, yet in 7 of 11 patients with lung metastasis was this found to be the case. Elevated CA 125 levels in primary lung cancer have been reported (Niwa & Shimokata, 1986; Van Niekerk et al., 1989; Kandylis et al., 1990; Kimura et al., 1990; Diez et al., 1991). In contrast, elevated levels of CA 125 in patients with bone tumours have been reported only in patients with disseminated metastatic disease; in primary bone cancer, e.g. osteosarcoma, chondrosarcoma, giant cell tumour and Ewing's tumour, CA 125 levels are normal (Shinozaki et al., 1992; Tsavaris et al., 1992). We have thus concluded that the increase in CA 125 in these breast cancer patients was caused by the metastatic infiltration of either the lung or the pleura: only in those patients with pleural involvement were CA 125 levels elevated; CA 125 levels in patients with no pleural involvement remained low. This hypothesis is further substantiated by reports indicating that CA 125 levels are elevated in patients with primary lung cancer predominantly when the pleura is affected (Niwa & Shimokata, 1986, Van Niekerk et al., 1989; Kandylis et al., 1990; Kimura et al., 1990; Diez et al., 1991).

The decision to change treatment in diffuse metastatic breast cancer is usually based on the progression of the most prominent metastases. If an increase in CA 125 levels in breast cancer patients is caused by pleural metastases (or lung metastases), then CA 125 measurement would permit assessment of the response of this site to treatment. If CA 125 levels drop during therapy of metastatic breast cancer then progression at another site should not necessarily lead to a change of the whole treatment. It could instead lead to a selective exchange of drugs. Since that would offer the development of new treatment strategies, this observation should be further investigated.

### References

BAST, Jr, R.C., KNAUF, S., EPENETOS, A., DHOKIA, B., DALY, L., TANNER, M., SOPER, J., CREASMAN, W., GALL, S., KNAPP, R.C., ZURAWSKI, Jr, V.R., SCHLOM, J., KUFE, D.W. & RITTS, Jr, R.E. (1991). Coordinate elevation of serum markers in ovarian cancer but not in benign disease. *Cancer*, **68**, 1758–1763.

COLOMER, R., RUIBAL, A. & SALVADOR, L. (1989). Circulating tumor marker levels in advanced breast carcinoma correlate with the extent of metastatic disease. *Cancer*, **64**, 1674–1681.

DE LOS FRAILES, M.T., STARK, S., JÄGER, W., HÖRAUF, A. & WILDT, L. (1993). Purification and characterization of the CA 125 tumor-associated antigen from human ascites. *Tumor Biol.*, **13**, 124–142.

DIEZ, M., CERDAN, F.J., ORTEGA, M.D., TORRES, A., PICARDO, A. & BALIBREA, J.L. (1991). Evaluation of serum CA 125 as a tumor marker in non-small cell lung cancer. *Cancer*, **67**, 150–154.

EINHORN, N., SJOVALL, K., KNAPP, R.C., HALL, P., SCULLY, R.E., BAST, Jr, R.C. & ZURAWSKI, Jr, V.R. (1992). Prospective evaluation of serum CA 125 levels for early detection of ovarian cancer. *Obstet. Gynecol.*, **80**, 14–18.

HARDARDOTTIR, H., PARMLEY, T.H. II, QUIRK, Jr, J.G., SANDERS, M.M., MILLER, F.C. & O'BRIEN, T.J. (1990). Distribution of CA 125 in embryonic tissues and adult derivatives of the fetal periderm. *Am. J. Obstet. Gynecol.*, **163**, 1925–1931.

JÄGER, W., ADAM, R., WILDT, L. & LANG, N. (1988). Serum CA 125 as a guideline for the timing of a second-look operation and second-line treatment in ovarian cancer. *Arch. Gynecol. Obstet.*, **243**, 91–99.

JÄGER, W., CILACI, S., MERKLE, E., PALAPELAS, V. & LANG, N. (1991). Analysis of the first signs of metastases in breast cancer patients. *Tumordiagn. u Ther.*, **12**, 60–64.

JÄGER, W., WILDT, L. & LEYENDECKER, G. (1986). CA 15-3 and CEA serum concentrations in breast cancer patients. In *Clinical Relevance of New Monoclonal Antibodies*, Greten, H. & Klapdor, R. (eds) pp. 167-173. Georg Thieme: Stuttgart.

KABAWAT, S.E., BAST, R.C., BHAN, A.K., WELCH, W.R., KNAPP, R.C. & COLVIN, R.B. (1983). Tissue distribution of a coelomic-epithelium-related antigen recognized by the monoclonal antibody OC125. *Int. J. Gynecol. Pathol.*, **2**, 275-285.

KANDYLIS, K., VASSILOMANOLAKIS, M., BAZIOTIS, N., PAPADIMITRIOU, A., TSOUSSIS, S., FERDERIGOU, A. & EFREDIMIS, A.P. (1990). Diagnostic significance of the tumour markers CEA, CA15-3 and CA 125 in malignant effusions in breast cancer. *Ann. Oncol.*, **6**, 435-438.

KIMURA, Y., FUJII, T., HAMAMOTO, K., MIYAGAWA, N., KATAOKA, M. & ITO, A. (1990). Serum CA125 level is a good prognostic indicator in lung cancer. *Br. J. Cancer.*, **62**, 676-678.

NIWA, Y. & SHIMOKATA, K. (1986). Diagnostic significance of cancer antigen 125, pancreatic oncofetal antigen, and carcinoembryonic antigen in malignant and tuberculous pleural effusions. *Jpn. J. Clin. Oncol.*, **16**, 3-8.

PEREY, L., HAYES, D.F., TONDINI, C., VAN MELLE, G., BAUER, J., LEMARCHAND, T., REYMOND, M., MACH, J.P. & LEYVRAZ, S. (1990). Elevated CA125 levels in patients with metastatic breast carcinoma. *Br. J. Cancer*, **62**, 668-670.

SECKL, M.J., RUSTIN, G.J.S. & COOMBES, R.C. (1992). CA 125 is not a useful marker in metastatic breast cancer. *Br. J. Cancer*, **66**, 875-876.

SHINOZAKI, T., CHIGIRA, M. & KATO, K. (1992). Multivariate analysis of serum tumor markers for diagnosis of skeletal metastases. *Cancer*, **69**, 108-112.

TSAVARIS, N., VONORTA, K., SARAFIDOU, M., BACOYIANNIS, C., MYLONAKIS, N., BEER, M., PAPAGRIGORIOU, D., KOUTSIOUBA-KAZAKOU, P. & KOSMIDIS, P. (1992). Comparison of tumor markers CEA, CA 125, CA 15.3 and prolactin levels in patients with advanced breast cancer. *Diagn. Oncol.*, **2**, 211-219.

VAN NIEKERK, C.C., JAP, P.H.K., THOMAS, C.M.G., SMEETS, D.F.C.M., RAMAEKERS, F.C.S. & POELS, L.G. (1989). Marker profile of mesothelial calls versus ovarian carcinoma cells. *Int. J. Cancer*, **43**, 1065-1071.